# A Transfer Learning Method for Detecting Alzheimer's Disease Based on Speech and Natural Language Processing

**DOI:** 10.3389/fpubh.2022.772592

**Published:** 2022-04-13

**Authors:** Ning Liu, Kexue Luo, Zhenming Yuan, Yan Chen

**Affiliations:** ^1^School of Public Health, Hangzhou Normal University, Hangzhou, China; ^2^Department of Mathematics and Computer Science, Fujian Provincial Key Laboratory of Data-Intensive Computing, Quanzhou Normal University, Quanzhou, China; ^3^Tongde Hospital of Zhejiang Province Geriatrics, Hangzhou, China; ^4^School of Information Science and Technology, Hangzhou Normal University, Hangzhou, China; ^5^International Unresponsive Wakefulness Syndrome and Consciousness Science Institute, Hangzhou Normal University, Hangzhou, China

**Keywords:** transfer learning, Alzheimer's disease, natural language processing, BERT, machine learning

## Abstract

Alzheimer's disease (AD) is a neurodegenerative disease that is difficult to be detected using convenient and reliable methods. The language change in patients with AD is an important signal of their cognitive status, which potentially helps in early diagnosis. In this study, we developed a transfer learning model based on speech and natural language processing (NLP) technology for the early diagnosis of AD. The lack of large datasets limits the use of complex neural network models without feature engineering, while transfer learning can effectively solve this problem. The transfer learning model is firstly pre-trained on large text datasets to get the pre-trained language model, and then, based on such a model, an AD classification model is performed on small training sets. Concretely, a distilled bidirectional encoder representation (distilBert) embedding, combined with a logistic regression classifier, is used to distinguish AD from normal controls. The model experiment was evaluated on Alzheimer's dementia recognition through spontaneous speech datasets in 2020, including the balanced 78 healthy controls (HC) and 78 patients with AD. The accuracy of the proposed model is 0.88, which is almost equivalent to the champion score in the challenge and a considerable improvement over the baseline of 75% established by organizers of the challenge. As a result, the transfer learning method in this study improves AD prediction, which does not only reduces the need for feature engineering but also addresses the lack of sufficiently large datasets.

## Introduction

Alzheimer's disease (AD) is a neurodegenerative and progressive disease that cannot be cured effectively (1). Mild cognitive impairment (MCI) is the early stage of AD. The study by the Lancet Public Health in 2020 found that the prevalence of dementia in people over 60 years old in China accounted for 6.04% of the population (approximately 1,507 ten thousand), and the number was 15.54% (3,877 ten thousand) for MCI cases (2). An epidemiological survey also found that a person's cost with AD in China was approximately $19,144.36 in 2015, while the total cost of the world's average level was $167,740 million, which was composed of $54,530 million (32.51%) direct medical cost, $26,200 million (15.62%) direct non-medical cost, and $87,010 billion (51.87%) indirect cost (3).

In the past 20 years, scholars have reported extensive studies on the relationship between the pathogenesis of AD and language fluency (4). They generally believed that mild word naming, retelling, hearing, understanding, and writing disorders already exist in the early stages of AD. One of the early signs of AD is an obvious decline in linguistic comprehension and expression form (5), and the linguistic manifestation of patients with AD usually includes the following:

1. Patients with AD talk less than ever before and are often silent, as they frequently forget the words they have just spoken, and have difficulty continuing with the topic that has just been discussed.
2. Sometimes, they are difficult to be understood with incoherent and repeated utterances.
3. They often call something the wrong name, for example, “watch” is regarded as “the clock on the wrist.”

New studies have found that before the onset of AD, the β-amyloid has already gathered in the brain about 5 to 10 and even 20 years ago. If AD can be diagnosed at an early stage (6), a series of behavioral therapies can be prescribed to slow the progress of the disease. However, an AD diagnosis is challenging in clinical medicine because of the subtle differences between patients with AD and healthy individuals in terms of brain structure and behavior. At present, some medical diagnosis methods, such as pathological examination, MRI, PET, and reliable biomarkers (e.g., amyloid ligand imaging and cerebrospinal fluid testing), are usually used. However, these diagnosis methods cannot be widely popularized because of their high cost and invasive nature. Therefore, there is an urgent need to develop a convenient, inexpensive, and non-invasive AD diagnostic approach by AI technologies, such as speech processing and NLP. In contrast to earlier studies with manual expert-wise feature extraction in this field, this study used a reliable deep learning model to automatically find suspicious AD symptom features from speeches. Specifically, a pre-trained distilBert language model (7) was used as a feature extractor to obtain the features of the input sentence or document, and a simple logistic regression classifier, which has a good effect on binary classification, was used to classify AD from normal controls. Owing to its strong deep semantic feature extraction competency and an accurate binary classifier, this combination can effectively improve the classification effect. In addition, a grid search strategy (8) was used to tune the parameters to obtain the best parameters of the model. The results show that this method worked better on ADReSS datasets (9) in 2020, with an accuracy of 0.88, which was significantly higher than the baseline and almost equivalent to the best performance on the challenge (10).

The main contributions of this study are as follows:

1. A simple and effective model of AD diagnosis based on transcripts without complicated expertise is designed and implemented effectively.
2. A novel model architecture that combines deep learning with machine learning is proposed, and the best performance on the ADReSS dataset is obtained.
3. Our proposed approach has the advantages of reliability, low cost, and convenience and can provide a feasible solution for the screening of AD.

## Related Works

Different technologies can be used to detect AD, such as molecular biomarkers combined with deep learning on gene expression datasets (11). However, we used transcripts combined with deep learning on speech datasets instead. Two approaches are mainly used in this field: machine learning with manual feature extraction based on expert knowledge and deep learning. Traditional machine learning algorithms have been widely studied with handcrafted features to predict AD. However, they have the disadvantage of lacking integrity, demanding good expertise, low accuracy, and poor portability. Moreover, these methods are generally applicable to a specific task scene. Once the scene changes, these manually designed features, and prior settings cannot be adapted to new scenes and need to be redesigned again; therefore, the portability of the model is not better overall. With the arrival of the deep learning paradigm, it has already become possible to extract high-level abstract features directly from transcripts that describe the distribution of datasets in low-dimensional manifolds internally. The advantage is that it can either extract input dataset patterns directly for both regression tasks or combine handcrafted features to the feature map of the input dataset without the certified professionals from the data source. Because language functions play an important role in the detection of cognitive deficits at different stages, the combination of NLP technology and deep learning provides an accurate and convenient solution for the detection of AD and MCI (12). In this study, a distilBERT model, which is a multi-layer perceptron with a self-attention mechanism, is used to extract deep semantic features; they are then passed through a strong binary classifier to recognize AD. The number of hidden layers is larger than that of traditional machine learning algorithms, thus, the model has a stronger semantic abstraction ability and classification performance, and the scalability is superior to traditional machine learning methods. Although the deep learning method does not need to extract features manually, it does not mean that we do not need to analyze manual features anymore, and the single deep learning model for diagnosing AD may perform better. Therefore, combining it with some conspicuous markers and a stronger classifier may improve the classification results, which we will discuss in the discussion section.

Several studies have investigated language and speech features for AD diagnosis (13) and proposed many signal processing and machine learning algorithms to detect AD and MCI (14). However, in this field, there are still lacking benchmark datasets against which different methods can be systematically compared. The ADReSS Challenge (9), a subset of the DementiaBank dataset (15), uses a balanced dataset of AD and healthy controls to recognize the disease. Manual feature extraction methods have a better interpretation for classification tasks, although there are unremarkable results. As a basic study on the ADReSS dataset, Luz et al. (9) used 34 linguistic features, such as total utterances, a type-token ratio, percentages of parts of speech, duration, MLU, and a word ratio, combined with linear discriminant analysis, and obtained the best accuracy of 0.75 on the test dataset. Acoustic features, such as emobase (16), the extended Geneva minimalistic acoustic parameter set (eGeMAPS) (17), minimal features (18), Computational Paralinguistics ChallengE (ComParE), (19), and multi-resolution cochleagram (MRCG) (20), only obtained an accuracy of approximately 0.5 on the classifiers used frequently. Balagopalan et al. (21) used two approaches for the binary classification of AD and normal controls, i.e., acoustic and text-based feature extraction and the bidirectional encoder representation (BERT) model. Finally, the BERT model obtained the best accuracy of 0.8332, which was better than that of the manual feature extraction method. Syed et al. (22) and Yuan et al. (23) achieved accuracies of 85.45 and 89.6% using acoustic and linguistic features, respectively. Syed et al. (22) used acoustic features, such as bag-of-acoustic-words and INTERSPEECH 2010 Paralinguistic Challenge feature sets [a low-dimensional version of ComParE (19)], and obtained an accuracy of 76.85%. Luz et al. (24) used a combination of phonetic and linguistic features without human intervention and obtained an accuracy of 78.87%. Most of these earlier studies were based on features designed by experts and were unable to learn more informative and discriminative features, so a relatively poor performance was obtained.

The latest deep-learning methods, such as convolutional neural networks (CNN), recurrent neural networks (RNN), and BERT, can achieve good performances by automatically extracting high-level features. Mahajan et al. (25) used part-of-speech (POS) tags and word embeddings (GloVe) as inputs on a CNN-long short-term memory (LSTM) model (26) and obtained the best accuracy of 0.6875. Then, they replaced unidirectional LSTM with bidirectional LSTM layers (27) and obtained the best accuracy of 0.7292. Orimaye et al. (28) used a deep neural network to predict MCI in speech. Different from our datasets, they used part of the Pitt corpus of the DementiaBank dataset, comprising 19 controls and 19 MCI transcripts. Fritsch et al. (29) enhanced *n*-gram language models to create neural network models with LSTM cells, and an accuracy of 85.6% was obtained to classify HCs and AD on the Pitt dataset. Pan et al. (30) used a glove word embedding sequence as the input, combined with gated recurrent unit layers and a stacked bidirectional LSTM to diagnose AD on the Pitt dataset. These models differ from our model because we used deep learning and machine learning classifiers instead. Similar to our method, the study (31) demonstrated that the combination of BERT_Large_ and logistic regression had the best performance in the classification problem. Different from our study, they used the Pitt DementiaBank dataset and data augmentation technology to enhance the classification performance and obtained a state-of-the-art (SOTA) accuracy of 88.08%.

Other tasks, except for the picture description task, can also be used to recognize AD. For example, Clarke et al. (32) used five different tasks to recognize AD, namely, conversation, procedural recall, picture description, narrative recall, and novel narrative retelling and obtained the best accuracy of 90% for AD vs. HC with linguistic features, combined with the support vector machine (SVM) model. In addition, many studies have used multimodal datasets to detect AD and MCI, and more accurate and differentiated information may be obtained from different models. Looze et al. (33) combined conversational features, neuropsychological testing, and structural MRI to explore temporal features, and a linear mixed model was used to diagnose AD, which differs from our corpus. They also found that slow turn-taking and slow speech are two useful factors for the early detection of cognitive decline. Martinc et al. (34) also used a multimodal approach to detect AD on the ADReSS dataset, using an active data representation approach (13), combining linguistic, acoustic, and temporal features and obtaining an accuracy of 93.75%. Jonell et al. (35) recorded participants' language, speech, motor signs, pupil dilation, thermal emission, facial gestures, gaze, and heart rate variability of 25 patients with AD and found that multi-modality improved clinical discrimination. Recently, the transfer learning model has been widely used to diagnose AD. For example, Laguarta et al. (36) presented an approach with multiple biomarkers, including sentiment, lung and respiratory tract, and vocal cords. They used the transfer learning model to learn the features from audio datasets and obtained an accuracy of 93% on the ADReSS datasets. Zhu et al. (37) used the transfer learning on the BERT model to detect AD with speech and text, achieving an accuracy of 89.58%. They also found that the text model was more discriminative than the speech model. Overall, strong representation learning ability and discriminative classifiers, multimodal information, and transfer learning are all effective factors in the accurate diagnosis of AD and MCI.

## Methods

### Transfer Learning

One of the challenges in AD prediction research is the lack of training data, which is important for a better understanding of language models with semantic and syntactic structures when they are implemented. Transferring knowledge from one model to another is called transfer learning, which is learning information from pre-trained datasets and then converting it into weights to transfer to another neural network. Therefore, we need not to train a neural network from scratch. It eliminates the need for target-specific large datasets using a model that learns a probable distribution for classification. The general flow of using a pre-trained model for classification consists of the following steps:

1. Training a general language model on a large dataset.
2. Fine-tuning a pre-trained language model on the target dataset.
3. Using a target-specific pre-trained language model for classification.

In this paper, we argue that the attention mechanism allows the model to focus on some parts of the transcripts for decision-making, which is suitable for AD diagnosis because it can capture specific markers related to AD. We used a pre-trained BERT model for text embedding, which converts original sentences or transcripts to 768-dimensional vectors. In the next part, we will describe the architecture of our model.

### Overall Classification Framework

The entire model architecture in this study mainly consists of two sections: the distilBert model (7) and the logistic regression classifier. The features transferred between the two models are 768-dimensional vectors, which are also embeddings for sentence classification.

**Figure 1:**
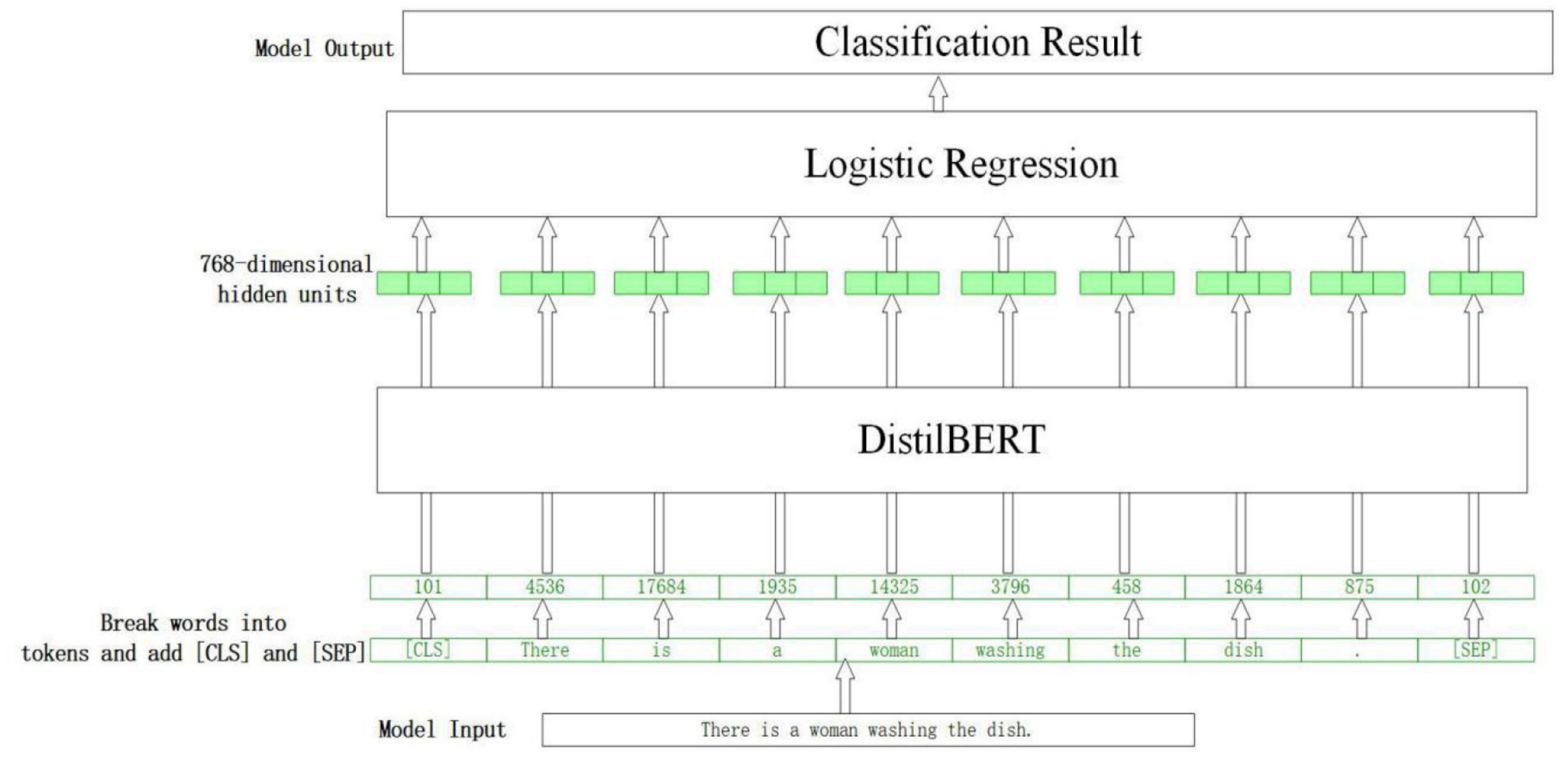
The logical architecture of the model.

Although BERT has become popular recently because of its excellent performance, the running speed with a hundred million parameters is a huge challenge for our computer system. Accordingly, we chose distilBert (7) developed by the team of Hugging Face, as an embedding feature extractor. It distills the BERT base from 12 layers to 6 layers and removes token-type embeddings and poolers. It can reach 60% of the faster speed and 40% smaller architecture but retains 97% language understanding capability of the BERT model (7). In this study, the distilBert model is used to extract deep semantic features, which are then passed to a logistic regression model to classify sentences. Specifically, the pretrained distilBert model is used as the feature extractor, the output layer of which is replaced by a logistic regression classifier for binary classification. The logical architecture of the model is shown in Figure 1. The embedding layer is a sentence or an entire transcript with a high-dimensional representation vector, and the classifier layer predicts the label of every embedded input. The main processes are as follows: Firstly, the words are divided into tokens using the distilBert tokenizer, and some special words are added to the text [i.e., (CLS) before the sentence and (SEP) at the end]. Then, the vocabulary table is searched from the pre-trained model to replace the tokens with the corresponding numbers taken into the DistilBert model and a 768-dimensional output vector is obtained. Finally, this vector is inputted into a logistic regression classifier, and the final binary classification result is obtained. The algorithm description of the entire process is presented below.

**Algorithm 1:** The process of our algorithm description.

1: Input: Dataset $D={\left\{\left(x_{i},y_{i}\right)\right\}}_{i=1}^{N}$; *x*_*i*_ is the input sentence; *y*_*i*_ is the corresponding label.
2: The load pre-trained model tokenizes a sentence by splitting the sentence into words or subwords and then pads all lists to the same size.
3: Use the distilBert model to train the dataset to obtain the embedding vector.
4: Put the embedding vector into the logistic regression model to classify the dataset.
5: Model evaluation.

DistilBert can capture long-distance dependencies by learning the global semantic message of input text thoroughly because it has some mechanisms, such as a multi-head self-attention and location code. It has excellent competence in feature extraction and semantic abstraction. The process is repeated six times and a 768-dimensional semantic feature vector is obtained, which is then input into a logistic regression model to get the final classification result. The transcripts in this study are a section of the description on a picture, the maximum length of which is no more than 500, so the length of word embedding is set as 500, considering speed and semantic completion.

### Grid Search

Grid search is a simple and widely used hyperparametric search algorithm fit for small datasets and can obtain the optimal value by searching all the points in the range. In this study, the GridSearchCV function in the scikit-learn tool, including grid search and cross-validation, is used to search for the best parameters of the logistic regression model. The grid search adjusts the parameters in sequence within a specified parameter range and then trains the model by using the adjusted parameters with the best performance in the validation set. The last score is the average of the *k*-fold cross-verification scores in the test set. Considering speed and accuracy, the search scope of the GridSearchCV function ranges from 0.0001 to 100, and the step is set as 20.

## Experiments

### ADReSS Datasets

**Figure 2:**
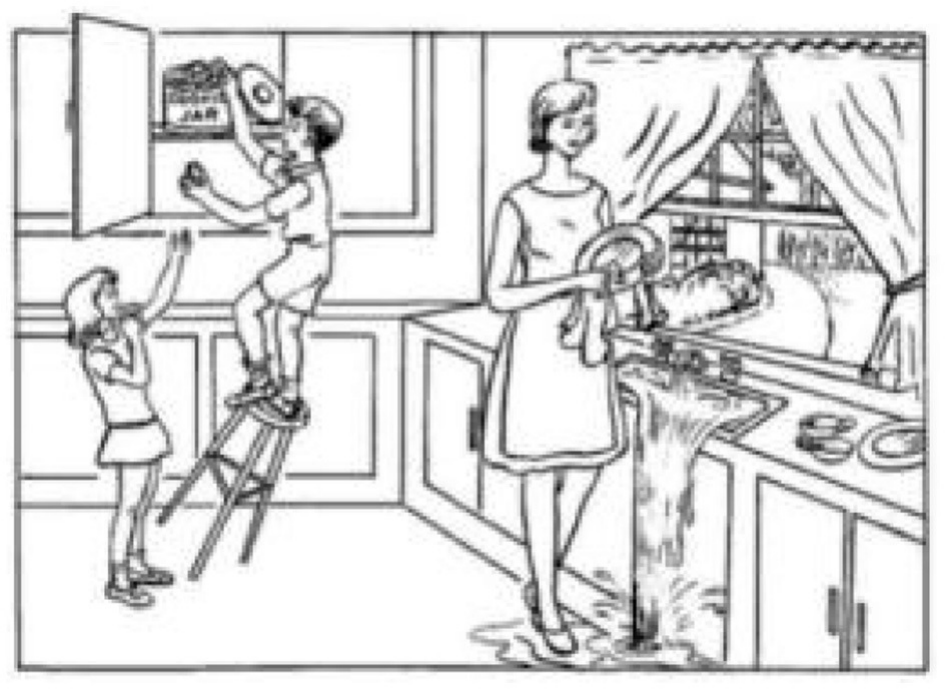
A picture of a Boston Cookie-Theft description task.

The study is a picture description task from the Diagnostic Aphasia Examination (38), and participants are asked to describe a picture (Figure 2) as detailed as possible. The datasets (9), including full-wave audio and corresponding transcripts with 78 AD and 78 normal controls, are divided into 108 training sets and 48 test sets by challenge, which has a balanced distribution for classes, gender, and age. An example of a transcript from the dataset is shown below.

*A boy and a girl are in a kitchen with their mothers. The little boy is getting a cookie for the little girl, but he is on a stool and is about to fall. The mother is washing dishes. She is obviously thinking of something else because the water pours out over the sink. She finished with some dishes. It seems to be summer because there are bushes. The window is open. There seems to be some kind of breeze because the curtains on the sill there blow. It must be fairly hot. The mother is in a sleeveless dress. The children are in short sleeve tops and have sandals. The little boy has tennis shoes. The mother obviously is unaware of what the children are doing. She will be aware of this shortly. How much more do you want to do?*

The age distribution of the two groups at different intervals is presented in Table 1. The average values and standard deviations of age and mini-mental state examination (MMSE) scores are shown in Table 2.

**Table 1:** The basic composition of the participants in every group.

	**AD (*****N*** **=** **78)**		**Non-AD (*****N*** **=** **78)**	
**Age interval**	**Male**	**Female**	**Male**	**Female**
50,55	2	0	2	0
55,60	7	6	7	6
60,65	4	9	4	9
65,70	9	14	9	14
70,75	9	11	9	11
75,80	4	3	4	3
Total	35	43	35	43

**Table 2:** The average and SD of age and MMSE.

	**Non-AD (** ***N** **=** **78)***		**AD (** ***N** **=** **78)***	
**Measure**	**AVG**	**SD**	**AVG**	**SD**
**Age**	66.56	6.60	66.79	6.83
**MMSE**	29.01	1.16	17.79	5.48

### Experiment Results

The experiment in this study was performed using the Windows 10 operating system. The computer was equipped with an Intel (R) Core I i5-6500 CPU @3.20 GHz, 3.19 GHz CPU, and 44. GB RAM. Library scikit learn was used to visit logistic regression, NumPy, and Pandas' libraries, and Python 3.6.13 was used as the programming language.

The experiment used the accuracy, precision, recall, and F1-score as indices to evaluate the performance of the model. Table 3 lists the relationship between the predicted and true classes. TP is a sample predicted to be positive. TN is a negative sample that is predicted to be negative. FP is a negative sample that is predicted to be positive. FN is a positive sample that is predicted to be negative. The formula for the metric index is as follows:


(1) $$\begin{array}{l} Accuracy=\frac{TN+TP}{TN+FP+FN+TP} \end{array}$$

(2) $$\begin{array}{l} Precision\;=\frac{TN}{TN+FP} \end{array}$$

(3) $$\begin{array}{l} Recall=\frac{TP}{TP+FN} \end{array}$$

(4) $$\begin{array}{l} F1-Score=\frac{2TP}{2TP+FP+FN} \end{array}$$

**Table 3:** Relationship between predicted class and true class.

	**True class**	
**Predicted class**	**Positive**	**Negative**
Positive	True positive(TP)	False positive (FP)
Negative	False negative(FN)	True negative (TN)

The parameters of the distilBert model are presented in Table 4. The champion of the ADReSS challenge obtained an accuracy of 0.896 by combining the Enhanced Language Representation with Informative Entities (ERNIE) model (39) and pause information in speech using acoustic align technology (10). We achieved 88% accuracy on the test set, which is almost equivalent to the SOTA result, and a 13% improvement over the baseline of 75%, established by the organizers of ADReSS (9). The champion used two models, acoustic and text, and combined the ERNIE model with discriminated markers to improve representation learning. We modified the model architecture of the distilBert model to achieve a strong classification performance using only text.

**Table 4:** Parameters of the distilBert model.

**Parameters**	**Value**
Epoch	1
DistilBatch_size	156
Pad_size	500
Pre-trained model	distilBert-base-uncased
Hidden_size	768

We used the popular models of BERT and ERNIE (39) for comparison. To check the influence of different classifiers with the DistilBert model, the CNN, random forest, SVM, and AdaBoost classifiers were also used for comparison with our logistic regression (LR) classifier. Table 5 shows that the LR classifier obtains the best performance. The LR is one of the simplest classifiers with a good performance in binary classification and has become a prior selection classifier in clinical diagnosis. For example, a study (31) demonstrated the superiority of the combination of BERT and LR models in the classification problem.

**Table 5:** The performance of different models.

**Model**	**Accuracy**	**Precision**	**Recall**	**F1-score**
Linear discriminant analysis (9)	0.625	0.60	0.75	0.67
DistilBert	0.48	0.51	0.48	0.48
ERNIE (39)	0.42	0.46	0.42	0.30
DistilBert +CNN	0.58	0.34	0.58	0.43
DistilBert+RF	0.79	0.79	0.79	0.79
DistilBert+SVM	0.625	0.629	0.625	0.622
DistilBert+Ada	0.73	0.73	0.73	0.73
ERNIE+Pause (10)^*^	**0.896**	**0.952**	0.833	**0.889**
DistilBert+ LR	0.88	0.88	**0.88**	0.87

* *ERNIE+Pause (10) is the model of a champion, distilBert +LR is our method, RF and Ada are the abbreviations of random forest and adaboost classifier, respectively*.

*The best performance in a column of measure*.

## Discussion

Pre-trained models are considered important and effective nowadays because they attempt to learn the features and structure of the language from large datasets and regulate the model effectively to perform best on new datasets by only updating a few parameters. Accordingly, our model was highly trained with the best initial parameters. The best performance indicates that our model has learned useful features for classification, which not only reduces the need for expert-defined linguistic features but also makes it possible for accurate, complex, and comprehensive features to be extracted from the dataset. The advantage of sentence embedding is that it considers the entire transcript and does not have any out-of-context word embedding layer, which converts every word into a vector representation, considering its context. The ADReSS challenge also includes MMSE evaluation, a detailed interactive exam to evaluate cognitive skills, including memory, language, delayed recall, and visuospatial. However, whether our model is suitable for the evaluation of MMSE scores needs to be further verified. In addition, the transcripts were annotated in CHAT format (40), which is convenient for manual feature extraction. We performed the experiment with and without annotation and found that the performance did not differ. Using automatic speech recognition (ASR)-generated transcripts directly without the need for further annotation, our method has more advantages than the manual feature extraction method.

Many studies have demonstrated that manual features, combined with the deep learning model, can improve the performance of the model, and manual features also provide a better interpretation, which is important for clinical diagnosis. For example, Looze CD et al. (33) found that the temporal characteristics of speech may reflect underlying cognitive deficits. Nasreen et al. (32) used linguistic features, such as pauses, overlaps, and dysfluencies, to detect AD on the ADReSS dataset. They obtained 90% accuracy and demonstrated the importance of dysfluency and pauses in detecting AD. The champion of the ADReSS challenge (10) combined deep learning with pauses and obtained SOTA accuracy of 89.6%, proving that pauses are important for AD diagnosis. Sadeghian et al. (41) extracted acoustic features, including pauses more than 5 s in duration, and obtained the best accuracy of 95.8%. Features, such as pauses, are important features that deep learning cannot learn effectively (i.e., cannot give enough weight for pauses), so the combination of both can improve the performance of AD detection. In clinical medicine, patients with AD often pause and cannot continue treatment. This is not only a memory decline problem but may also be related to some language function obstacles caused by brain damage. A successful computer model can guide doctors to focus more on the early clinical symptoms of patients with AD, such as pauses and dysfluency. The largest limitation of our study is the difficulty to interpret the performance of a model with so many parameters (42). That is, our model cannot understand the reason for a wrong verdict, but we can identify the words that the network has paid more attention to in the case of a correct prediction. This function is particularly useful because such an interpretation can reveal the important linguistic attributes of patients with AD, which can help in speech therapy and communication with patients with AD.

The practice of pre-trained and fine-tuning paradigms has achieved excellent performance in many downstream tasks. In recent years, research in academia and industry has indicated that the pre-trained model is developing in a larger and deeper direction. However, there are still some problems that need to be solved in large models, such as the dataset quality, huge training energy consumption, carbon emission problems, and a lack of common sense and reasoning ability. These problems should be addressed in future studies.

## Future Works

In the future, we will focus on the following two directions for AD diagnosis.

Implicit sentiment analysis is an expression that does not contain any polarity markers but can still convey a clear human awareness sentiment polarity in the context; it exists widely in the recognition of aspect-based sentiments (43). For example, the comment “The waiter poured water on my clothes and walked away” contains no opinion words but can be interpreted as clearly negative toward “the waiter”; some sentences, such as “the service of the hotel is great,” “the food of the restaurant is delicious,” contain obvious sentimental words that neural network can give enough weights for the words of “great,” “delicious,” but the non-sentiment-related aspects of such words are often ignored by the model. The transcripts used for the AD diagnosis of spontaneous speech contain no polarity markers; however, most previous studies in this field generally pay little attention to implicit sentiment expressions. The study (44) used supervised contrastive learning to capture implicit sentiment using an advanced method. That is, the expressions with the same sentiment polarity were pulled together, and those with different sentiment orientations were pushed apart. In the future, we will focus on implicit sentiment analysis for AD diagnosis using a contrastive learning method.

One of the most popular language models is the multilingual one. With a proper multilingual model, the problem of lacking large datasets can be addressed by transferring the knowledge of AD prediction from another language in which a large dataset is available, which is similar to the approaches proposed by Fraser et al. (45). Only in this manner can the need for a target task be addressed for expert-defined linguistic features. In the future, we will commit to improving multilingual AD recognition using cross-lingual transfer learning, including the multilingual BERT and transformer models.

## Data Availability Statement

The datasets presented in this study can be found in online repositories. The names of the repository/repositories and accession number(s) can be found in the article/supplementary material.

## Ethics Statement

Written informed consent was obtained from the individual(s) for the publication of any potentially identifiable images or data included in this article.

## Author Contributions

ZY designed the research. KL analyzed the data and interpreted the analysis. NL and YC wrote the main manuscript text and revised it carefully. All authors reviewed and approved the final manuscript.

## Funding

This research was funded by Natural Science Foundation of Zhejiang Province (Grant Number LQ22H090002), the initial fee for introducing doctoral research of Natural Science Foundation of Zhejiang Provincial (Grant Number LGF20F020009) and the 4th Graduate Student innovation and Entrepreneurship Competition of Hangzhou Normal University.

## Conflict of Interest

The authors declare that the research was conducted in the absence of any commercial or financial relationships that could be construed as a potential conflict of interest.

## Publisher's Note

All claims expressed in this article are solely those of the authors and do not necessarily represent those of their affiliated organizations, or those of the publisher, the editors and the reviewers. Any product that may be evaluated in this article, or claim that may be made by its manufacturer, is not guaranteed or endorsed by the publisher.
